# Beyond “getting drugs into bodies”: social science perspectives on pre-exposure prophylaxis for HIV

**DOI:** 10.7448/IAS.18.4.19983

**Published:** 2015-07-20

**Authors:** Judith D Auerbach, Trevor A Hoppe

**Affiliations:** 1 Center for AIDS Prevention Studies, Department of Medicine, School of Medicine, University of California, San Francisco, CA, USA; 2 Department of Criminology, Law & Society, University of California, Irvine, Irvine, CA, USA

**Keywords:** PrEP, social science, HIV prevention, sexuality, risk compensation

## Abstract

Social scientists have much to contribute to the analysis of the real and potential contribution of pre-exposure prophylaxis (PrEP) to HIV prevention around the world. Beyond just a matter of clinical efficacy and getting pills into people's mouths, PrEP raises a number of important social-psychological questions that must be attended to in order to translate biomedical and clinical findings into uptake of PrEP among enough people at risk of HIV infection to produce population-level effectiveness. PrEP is a dynamic phenomenon with “dialectical” attributes that invite both optimism and cynicism as a desirable and effective HIV prevention strategy. PrEP disrupts traditional notions of “safe” and “unsafe” sex; it confers on its users a level of agency and control not generally achieved with condoms; and it affects sexual practices and sexual cultures in meaningful ways. As these dynamics play out in different contexts, and as new modes of PrEP administration emerge, it will be important for social scientists to be engaged in assessing their impact on PrEP implementation and effectiveness.

## Introduction

Among the HIV biomedical, clinical and advocacy communities, discussions of pre-exposure prophylaxis (PrEP) have largely focused on two questions: Is it clinically effective? and What are the structural and policy factors that impact its effectiveness when implemented? But, PrEP raises a number of other important social-psychological questions that also must be attended to in order to translate biomedical and clinical findings into uptake of PrEP among enough people at risk of HIV infection to produce population-level effectiveness. Social science has much to contribute in this regard.

Social scientists take as their starting point that PrEP is a dynamic phenomenon that is more than just a pharmacologic intervention – that is, getting PrEP to “work” is more complicated than simply “getting drugs into bodies.” Rather, PrEP embodies a range of interacting physiological, psychological and social realities that together affect not only an individual's risk or avoidance of HIV infection but also relationship dynamics, sexual cultures and social arrangements that have influence beyond HIV. We explore some of this dynamism and the issues it raises for further understanding of the role of PrEP in HIV prevention and to make the case that social science perspectives are essential as further implementation of PrEP ensues.

## The dynamic nature of PrEP

PrEP emerges in, and itself effects, a dynamic situation. In the context of combination HIV prevention, PrEP provides another method in the ever-evolving constellation (or “toolbox”) of evidence-based prevention strategies. It enhances the repertoire of choices individuals can make about how best to protect themselves from acquiring HIV, taking into account the realities of one's life, the nature of one's sexual and drug-using practices and relationships, and personal preferences about behavioural and technological “interventions.”

Perhaps most importantly, PrEP's demonstrated efficacy among gay and other men who have sex with men (MSM) and transgender women [1], heterosexual men and women [2, 3], and men and women who inject drugs [4] disrupt traditional notions of “protected” and “unprotected” sex, and of “risky” and “safe” sex and drug use [5–7] – notions that have been institutionalized in public health and community (especially gay community) discourse and practice for the past three decades. (As a case in point, PrEP's disruptive effect, alongside other advances in using antiretroviral (ARV) drugs for HIV prevention, recently helped to spur the U.S. Centers for Disease Control and Prevention (CDC) to stop using the term “unprotected sex” to refer to sexual intercourse without a condom [8].)

The promotion of ARV-based prevention approaches – whether “treatment as prevention,” “pre-exposure prophylaxis” or “post-exposure prophylaxis” – imbues the person taking ARV with a responsibility to care for his/her health as well as that of others [9]. Throughout the course of the HIV epidemic, the collective responsibility for preventing HIV has shifted from the promotion of condom use by HIV-negative persons to recommending that HIV-positive individuals begin taking ARV early in the course of their infection. Efforts to promote PrEP could again shift the responsibility for prevention back towards HIV-negative individuals [10], underscoring the dynamic ways in which individuals (and couples) interact with drugs and the drugs change their realities.

## The dialectics of PrEP

When PrEP first emerged on the HIV prevention landscape, much of the popular discourse surrounding it was framed in a binary and oppositional fashion, that is, either PrEP holds the promise to ending the HIV pandemic or PrEP is an insidious strategy that will exacerbate HIV epidemics and attendant social ills [11]. Many social scientists argue instead that PrEP as a technology is not inherently “good” or “bad” – it has both positive and negative potentialities simultaneously and produces something new entirely as a result of the dynamic tension between them [6]^.^ This dialectic can be seen in a number of areas explored below.

### Efficacy

Evidence from key clinical trials has shown that, if taken daily as prescribed, oral Truvada for PrEP is a highly efficacious HIV prevention strategy. It may reduce HIV acquisition by more than 90%, placing it right next to male latex condoms and access to sterile syringes as the most efficacious HIV prevention methods available today. But, data from the same clinical trials and others [12] indicate that most participants did not take oral PrEP as prescribed. Although adherence observed in clinical trials is likely to vary considerably from levels in the “real world,” trial results suggest that implementation programmes may need to greatly increase adherence levels in order to maximize the likelihood that PrEP will have a population-level impact.

Furthermore, although treatment-resistant mutations have not been witnessed in clinical trials as of yet, there is some concern that low adherence levels create the potential in individuals with partial adherence to develop resistance to certain classes of ARV, should they become HIV infected (and to transmit those strains onward). However, especially in light of recent data suggesting that even intermittent use of PrEP can be highly effective at preventing infections [13], evidence to date does not support this argument.

### Agency and control

PrEP also has the potential to confer agency and control on HIV-uninfected persons who heretofore have had to depend on willingness of partners to use condoms or ARV as their primary prevention strategies [14, 15]. The possibility of using PrEP without the knowledge of the other partner is a very important development for anyone who needs an HIV prevention method that can be used surreptitiously, as has been argued for microbicides, particularly for women [16, 17].

Relatedly, PrEP in its current form as a once-daily pill is not coitally dependent, so individuals can take it at any time during the day they wish and not have it interrupt or interfere with any particular sexual episode (i.e., before, during or after intercourse). This unobtrusiveness, in combination with the relief of knowing one is protected from HIV, confers some level of control on the PrEP user. It also imbues PrEP with the potential to enhance sexual pleasure and fulfilment [18]. This should continue to be the case when other methods of PrEP administration (such as injectables and vaginal rings) become available, as these are also not coitally dependent and, to a great extent, can be used without others necessarily knowing [19].

However, although PrEP has this potential to confer agency and control in the user, it is not that simple. The only currently available PrEP method – Truvada – is the same pill that is used to treat HIV-positive persons. In many settings where HIV-associated stigma is high, being seen with “the little blue pill” (Truvada) implies being HIV infected, regardless of how a person on PrEP attempts to explain its use for prevention [18] The associated stigma may be a big disincentive for HIV-uninfected persons to take up PrEP [18, 20, 21]. Moreover, broad cultural and institutional stigma associated with sexuality, substance use and HIV may militate against access to PrEP services and engagement in related care in many settings.

### PrEP and sexuality

One of the most controversial aspects of PrEP is that of “risk compensation” [5, 15]. The fear is that PrEP users will decrease condom use or substitute PrEP for it, thereby enhancing the potential for increased sexually transmitted infections (STI), if not HIV transmission. But, arguing against PrEP based on the fact that it does not protect against other STIs is problematic in at least two ways. First, PrEP taken correctly confers as much, if not more, protection from HIV than do male latex condoms. Thus, if HIV prevention is the primary goal of PrEP, aversion of new HIV infections ought to be the outcome of relevance, and PrEP should be acknowledged as highly successful in this regard. Second, many people most at risk of acquiring HIV are the very ones who simply are not using condoms and who are, therefore, at risk of acquiring both HIV and other STIs [22]. PrEP may be an important way for these individuals to at least prevent the more dangerous disease – HIV – and, therefore, it ought not to be rejected because of what else it does not avert. Moreover, evidence to date from long-term follow-up from PrEP clinical trials and from open-label studies indicates that “risk compensation” has not occurred among either gay and other men who have sex with men (MSM) or heterosexuals using PrEP [23–25]. Although there has been incidence of hepatitis C and other STIs among those who use PrEP, no evidence yet exists to suggest that PrEP users experience increased rates of STI as compared to their at-risk counterparts.

Among gay men, fear about abandoning condoms goes beyond public health concerns and touches core issues in sexual culture. On the one hand, PrEP is creating a new form of “safe sex” that does not rely on barrier prevention methods (such as latex condoms), allowing its users to experience barrier-free intimacy without fear of contracting HIV. On the other hand, the potential for PrEP to confer a new level of agency, control and pleasure in sexual relations, in combination with the fears of “risk compensation,” has fuelled a new sexual moralism, particularly within gay communities. Early public debates in the gay community were framed around a controversial online essay that labelled PrEP users as “Truvada whores” [26–28]. Intended as a stigmatizing label, activists reappropriated the term as a message of pride and launched a PrEP campaign with T-shirts starkly emblazoned with the phrase [29]. But, the cultural association of PrEP with a kind of “unbridled” sex may have contributed to its slow uptake [7].

Scholars have noted that the so-called “PrEP wars” resemble debates over birth control for women [30]. Many of the issues raised in argument against PrEP are identical to those invoked against female contraception, namely, cost, safety, the potential impact on sexual behaviour and the potential for unforeseen health risks associated with long-term use. These issues are not new or specific to HIV, and concern about them is largely driven by a version of sexual morality – that sex is taboo, that it is self-destructive and that sexual pleasure is sinful and disgraceful [7, 31].

Despite its sociocultural baggage, PrEP already has begun to reshape the sexual landscape in many communities. For example, online sexual hook-up websites for gay men now offer an expanding variety of options for characterizing one's HIV status, with at least five HIV status options apparently now in circulation: *HIV-negative; HIV-negative and on PrEP; HIV-positive and not on treatment; HIV-positive with an undetectable viral load* and *I don't know* [32]. These provide users of the website with information they use to guide sexual practice and to imagine and define their sexual communities. The sociocultural implications of this shift are significant, as there are signs that the HIV-positive/negative binary that has persisted since the advent of the HIV test in the early 1980s may be eroding [33–35]. This trend has implications for serodiscordant relationships, as PrEP offers a way to safeguard health while preserving the relationship and promoting intimacy [36]. For heterosexual couples, PrEP provides a relatively easy way (as compared to previously favoured techniques, such as sperm washing or intrauterine insemination) to facilitate pregnancy without risking HIV transmission [37]. In short, in the context of PrEP, the risk of seroconversion is no longer the significant obstacle it has been to serodiscordant intimacy and partnership.

### The “substitutive” nature of PrEP

Beyond “risk compensation,” there are other concerns about the ways in which PrEP may become a substitute for extant HIV prevention and treatment strategies, with resultant ill effects. At a policy level, there is some concern that governments and other payers will shift resources from behavioural counselling, HIV testing, condom promotion, social support and harm reduction services to PrEP programmes, with negative consequences for certain populations [38]. Some argue that PrEP likely will not benefit those most in need – including people who use drugs and/or have mental health problems, or who experience instability in their housing situations – because of cost and adherence issues, and, as such, PrEP use might enhance existing HIV-associated disparities [39]. Still others have argued that implementing PrEP in low-resource environments would be unethical because it would threaten to shift resources away from treatment [40, 41]. These critics raise important issues of equality and justice. Their arguments point to the current institutional (financial and policy) context for funding HIV prevention in general (which in many settings is dismal) that result in the counterpoising of ART use for primary prevention and ART use for treatment.

Taking all these considerations together, it is clearly the case, as Peter Aggleton has noted, that “PrEP is an HIV prevention strategy that may be useful to some people in some contexts some of the time” [42]. If and how it is used, and with what potential effect, will vary across individuals, social groups, populations and social, political and economic systems. It is important that social scientists investigate how all this occurs and plays out globally over time, and with what consequences for individuals and societies.

## Conclusions

The promise of PrEP is not yet being fully realized, in part because not enough is being done to understand the social dynamics of the prevention strategy. From a clinical standpoint, adherence may appear to be the problem that stands between PrEP and its potential impact. But from a sociological perspective, there is a much richer set of issues that shape PrEP and its social and clinical significance. PrEP's efficacy and effectiveness – alone and in combination with other HIV prevention methods – are not simply a function of “getting drugs into bodies.”

Beyond adherence, implementing PrEP will require understanding how individuals and communities comprehend it. Do they believe it is effective? Do they trust the agencies and individuals promoting it? Do they think that they have access to it and can afford it? And, perhaps the most significant question is whether potential PrEP users understand themselves to be at risk of acquiring HIV, and, if so, whether that risk is sufficient for them to proactively engage in HIV prevention.

Beyond simple use or non-use of PrEP, social research can help us understand what meanings people assign to it. Is PrEP another “little blue pill” that they associate with “recreational” sex? Is it a symbol of love or intimacy with their partner? Is it a marker of the rich or elite who have the “privilege” to use it? PrEP's symbolic life will become just as important as its clinical efficacy in shaping how communities engage with it. Social science methods can help evaluate what impact PrEP has on sexual (and drug using) practices and cultures – beyond merely “risk compensation.” For example, “neg+PrEP” is fast becoming a new identity for gay men using online hook-up applications. How does this self-proclaimed status shape one's interactions with other men? Does taking PrEP encourage some users to explore sexual practices (such as receptive anal intercourse) that they once avoided for fear of infection? These possibilities are not just merely a matter of “risk”; they shape sexual cultures and thus have important sociological implications.

Perhaps most importantly, social science can help us reveal what PrEP tells us about the state of our public health infrastructure and the organized AIDS response community. Nearly three years after PrEP's FDA approval, the drug remains relatively underutilized. What does the slow-paced embrace of PrEP by health departments, medical providers, HIV/AIDS advocates and AIDS service organizations tell us about institutions of public health and medicine and AIDS advocacy? These socio-structural questions provide important pathways for understanding PrEP as an object circulating in social space more generally.

As interest intensifies in implementing and scaling-up PrEP in both clinical and community settings, as it now appears to be doing, the potential for social science research on PrEP – and the need to incorporate its findings – has never been greater.
